# *Incadendron *: a new genus of Euphorbiaceae tribe Hippomaneae from the sub-Andean cordilleras of Ecuador and Peru

**DOI:** 10.3897/phytokeys.85.14757

**Published:** 2017-08-31

**Authors:** Kenneth J. Wurdack, William Farfan-Rios

**Affiliations:** 1 Department of Botany, MRC-166, National Museum of Natural History, Smithsonian Institution, P.O. Box 37012, Washington DC 20013-7012, USA; 2 Department of Biology, Wake Forest University, Winston-Salem, NC 27106, USA

**Keywords:** Anatomy, ecology, Euphorbiaceae, Hippomaneae, *Incadendron*, ptyxis, taxonomy

## Abstract

*Incadendron esseri* K.Wurdack & Farfan, **gen. & sp. nov.**, from the wet sub-Andean cordilleras of Ecuador (Cordillera del Cóndor) and Peru (Cusco, Oxapampa) is described and illustrated. This recently discovered large canopy tree with a narrow elevational range presents an unusual combination of rare morphological characters in Hippomaneae including mucilage-secreting sheathing stipules, conduplicate ptyxis, and large, woody fruits. The broader significance of these characters in Hippomaneae is discussed. The morphology and anatomy of *Incadendron* were investigated, highlighting its fruit similarities with Guiana Shield endemic *Senefelderopsis*, and the systematics value of ptyxis variation, which remains poorly studied for the family.

## Introduction

Euphorbiaceae contains about 330 genera and 6300 species, and new genera continue to be added both through taxonomic adjustments to non-monophyletic groups discovered during molecular phylogenetic studies (e.g., *Karima*, Cheek et al. 2016), and more rarely through their discovery as recently collected novelties (e.g., *Gradyana*, Athiê-Souza et al. 2015; *Tsaiodendron*, Zhou et al. 2017). One such novelty, described herein, has been relatively well collected over the past 15 years due to intensive floristic and ecological studies in several sub-Andean cordilleras of Ecuador and Peru. These wet, mid-elevation mountains between the main Andean chain to their west and Amazonian lowlands to their east are rich in plant and animal endemism, especially in the Cordillera del Cóndor (Neill 2005, Ulloa Ulloa and Neill 2006), which contains the northern part of the known range of this plant and has floristic ties to the Guiana Shield. However, this region is relatively sparse in Euphorbiaceae endemism and a new genus is noteworthy. Based on morphological features, the Andean plant was quickly determined to be an undescribed member of Euphorbiaceae subfamily Euphorbioideae, and affiliated with tribe Hippomaneae. The shared diagnostic features include exudate of white latex, indument lacking, staminate bracts glandular at their base, perianth small in both pistillate and staminate flowers, petals absent, and staminate flowers inclinate in bud.

Euphorbioideae contains 40–45 genera (Esser 2001, Radcliffe-Smith 2001, Webster 2014; plus newly described *Gradyana*, Athiê-Souza et al. 2015) grouped in 3–5 tribes, and includes about 30 genera with New World species. Most of those 30 genera belong to tribe Hippomaneae and are wholly restricted to the neotropics, although a few have North American and/or Old World species. The taxonomy of the subfamily is difficult in regard to tribal and some generic circumscriptions, and a reclassification is needed within a phylogenetic framework (Wurdack et al. 2005). Most of the taxonomic problems relate to Hippomaneae, and distinctions among its many small-flowered, constituent genera are especially challenging. Such difficulties were found to be the case when considering the taxonomy of the Andean plant. Molecular phylogenetic placement within a well-sampled 4-gene phylogeny of subfamily Euphorbioideae (Wurdack, unpublished) establishes it as a member of subclade H1 of the hippomanoids (i.e., the clade of three tribes comprising paraphyletic Hippomaneae with embedded Hureae and Pachystromateae; Wurdack et al. 2005), although its divergence is deep and resolution is poor as to its closest relatives. The lack of assignment to an existing genus using morphological and molecular evidence indicates it deserves recognition as the novelty described below.

## Taxonomic treatment

### Incadendron

Taxon classificationPlantaeMalpighialesEuphorbiaceae

K.Wurdack & Farfan gen. nov.

urn:lsid:ipni.org:names:77165359-1

#### Diagnosis.

Differs from other members of Euphorbiaceae by its combination of exudate of white latex, indument lacking; leaves coriaceous, with marginal glands, ptyxis conduplicate; stipules, large, sheathing, mucilage-secreting, deciduous; inflorescences leaf-opposed, spicate, with solitary pistillate flowers and numerous 3-flowered, glandular staminate cymules; flowers apetalous with 3 sepals, staminate flowers with 3 stamens; fruits large, woody, dehiscent; and seeds dry, ecarunculate.

#### Type species.

*Incadendron esseri* K. Wurdack & Farfan.

#### Description.

Monoecious, glabrous trees. Latex white. Leaves alternate, petiolate, stipulate, simple, entire, glandular along margin near base, base minutely auriculate, penninerved, coriaceous, ptyxis conduplicate; stipules large and sheathing, entire, mucilage-secreting, eglandular, deciduous; petioles eglandular. Inflorescences terminal, solitary, appearing leaf-opposed, subtended by stipuliform bracts; pistillate flower 0–1, basal, bracteate; staminate flowers distal in numerous 3-flowered cymules subtended by verruculose bract and glands, bracteoles absent. Staminate flowers inclinate in bud and later erect, pedicellate; sepals 3, shortly connate; stamens 3, filaments short, free, anthers basifixed, extrorse, 2-thecate, longitudinally dehiscent; petals, pistillodes, staminodes, and disc absent. Pollen subprolate in equatorial view, 3-lobate in polar view, 3-colporate, margo present, tectum perforate. Pistillate flowers pedicellate; calyx 3-lobed, eglandular; ovary smooth, 3-locular, ovules 1 per locule, styles long; stigmas 3, undivided, eglandular; petals, staminodes, and disc absent. Fruit long-pedicellate, subglobose, smooth, dehiscing septicidally into 3 mericarps; pericarp dry, woody, thick; septa of mericarps with single or bifurcated vascular strand; columella alate, persistent. Seeds 3 per fruit, ellipsoid, smooth, ecarunculate.

### Incadendron esseri

Taxon classificationPlantaeMalpighialesEuphorbiaceae

K.Wurdack & Farfan sp. nov.

urn:lsid:ipni.org:names:77165360-1

Figure 1

#### Type.

PERU. Cusco: La Convención, Districto Quellouno, Abra de Yavero, 12°28'43"S 072°29'00"W, 2301 m, 24 Sep 2007 (fl, fr green fide label), *G. Calatayud, I. Huamantupa, E. Suclli, & R. Ayerbe 4711* (holotype: USM; isotypes: AMAZ, CUZ, HUT, MO-6669029, MOL, US-3679263).

#### Description.

*Trees* 6–26 m tall, to at least 56.7 cm dbh, trunk bark thin, monoecious; flowering and fruiting branchlets with leaves present, branching in pairs, leafy stems 1.5–2 mm dia., internodes 1–1.5 cm apart, branchlet bark smooth with scattered leaf and stipule scars, pith soft. *Exudate* present, white latex, watery. *Indument* absent. *Leaves* alternate, petiolate, stipulate, simple. *Stipules* free, paired, overlapping to sheath terminal bud, lanceolate, 10–13 × 3–4 mm, tip acute; base cordate, slightly asymmetric, with rounded free lobes extending to 1 mm below central point of attachment; eglandular, margins hyaline, deciduous before new leaf has fully expanded (abscised before leaf is 1/3 of mature size), after abscission leaving elliptic to reniform scars 1.3–1.7 × 0.4–0.5 mm. *Petioles* 9–20 × 1–1.8 mm (dia. mid-length), slightly flared at base, adaxially canaliculate, groove shallow and wide at petiole base (proximal) then narrowed mid-length and finally deep and wide at distal apex where shoulders of grove support minutely auriculate leaf base, petiolar glands absent. *Leaf blades*: laminar size class microphyll to notophyll, blade 6.7–13 × 2.9–3.8 cm, length:width ratio 1.97–2.89:1 (mean = 2.49, SD = 0.212, n = 40, 2–3 mature leaves each from 14 collections), symmetrical, elliptic to slightly obovate, apex acute to rounded, often minutely retuse, base acute and minutely auriculate at point of attachment; margin with distinct smooth edge ca 0.1 mm wide, slightly revolute with more pronounced inward rolling at leaf base; marginal glands up to 12 per side with 2–4 larger ones consistently near base then sparse and progressively smaller distally, glands embedded in leaf margin (those at base hidden or appearing abaxial due to rolling of margin) and often appear perpendicular to blade, 0.2–1 × 0.1–0.5 mm, narrowly elliptic, surface slightly sunken, smooth; apex of midvein (where terminating at margin zone) minutely apiculate or thickened with globular mass 0.2 mm dia.; lamina coriaceous, adaxial surface of new leaves glossy, abaxially appearing smooth; ptyxis conduplicate, halves of young blade folded tightly together along adaxial surface; embedded laminar glands absent. *Venation* pinnate, brochidodromous; secondaries (10)13–17(19) pairs, spacing uniform, vein angle uniform to decreasing proximally, (10)20–30(40)°, decurrent attachment to midvein; intersecondaries frequent, parallel to secondaries; intercostal tertiaries reticulate; primary to tertiary venation prominulous on both surfaces. *Inflorescence* bisexual or staminate, solitary, terminal but appearing leaf-opposed, spicate thyrse, 5–12 cm long including 0.9–1.8 cm peduncle and distal rachis; in young bud protected by adjacent sheathing leaf stipules and bracts; bracts 2, free to shortly connate, stipuliform, inserted at start of fertile part (i.e., just below pistillate flower), 7 × 3.5 mm, rarely with 1–2 glands at base, cauducous. *Flowers* unisexual, lacking petals, disc, staminodes, or pistillodes. *Staminate partial inflorescence* a lax spiral of numerous cymules, each cymule on a tissue pad bearing a bract and subtended by glands; cymule bracts ca 0.5 (free portion) × 1 mm, widely acute to rounded, verruculose, persistent, when young forming protective scale-like sheath around staminate subinflorescence and inclinate buds; glands 4–5(6) in row or cluster per side of bract, 0.5–0.6 × 0.3–0.4 mm, disc-shaped or prismoidal when tightly abutting each other, face concave, yellow-orange in life; flowers 3 per cymule, central flower precocious and senescent well before laterals; bracteoles absent. *Staminate flowers* erect at anthesis; pedicels 3 × 0.3 mm for central flower (laterals seen only in bud), articulated at base; sepals 3, connate to 0.2 mm, widely rounded, 0.5 × 0.7 mm; stamens 3, free; filaments distinct, shorter than anthers, to 0.3 mm long; anthers 0.6 × 0.6 mm, 2-thecate, basifixed, exserted slightly through sinus between sepal lobes, dehiscent through longitudinal slits to 0.3 mm long, slit margins slightly recurved at anthesis; connective tip acute, barely protruding beyond thecae, not elaborated; yellow in life. *Pollen* polar:equatorial ratio (1.13)1.16–1.25:1 (based on SEM), tectum perforations smaller near apertures. *Pistillate flower*: (0)1, basal, pedicellate; pedicel 3.5–4.5 × 0.8–1 mm; pistillate bracts (1)2, at base of pedicel (shortly distal to stipuliform inflorescence bracts), 1–4 × 0.6–1 mm, elliptic to lanceolate, cauducous, leaving scars 0.3 × 0.2 mm; glands absent; flower 6–9 mm long; sepals 3, connate to 1 mm, broadly acute, 1.7–3.5 × 1.8–1.9 mm, margins hyaline; ovary 3-locular, ca 2 × 2 mm, top tapering and barely distinguished from start of styles; placentation apical pendulous with single ovule per locule; styles connate into column ca 5 × 0.9–1 mm; stigmas 3, free, undivided, eglandular, ca 3 mm long, erect in bud, recurved to loosely coiled at anthesis, surface minutely papillose. *Infructescence* axis 23–40 mm long, consisting of peduncle 11–20 × 1.3–1.5 mm (dia. mid-length) and pistillate flower pedicel 10–20 × 1–1.3 mm (dia. mid-length and distinctly thinner than peduncle), prominent scars where staminate partial inflorescence and bracts abscised. *Fruit* subglobose, trigonous, 20 × 20 mm; ventral (septal) sutures sulcate; dorsal (loculicidal) sutures smooth when fresh, becoming ridged when dry; apex with woody beak ca 3 mm tall, sepals and stigmas deciduous; mericarps equal, 2-valved, splitting septicidally then loculicidally to release seeds. *Pericarp* 3–4 mm thick (equatorial at dorsal suture); dried exocarp thin, 0.2 mm mid-mericarp to 1 mm at ventral septal sutures and 2 mm at base, loosely adherent (can be easily peeled off dry specimens) to woody mesocarp on dehiscence, prominulous (likely drying artifact) veined with one primary vein descending from apex per valve, lower vein orders reticulate, major venation tracking embossed ridges on woody mesocarp; mericarp valves (cocci) barely twisted when dehisced, remaining attached together via basal triangle 3–5 × 6–7 mm (width at base); septa of mericarps woody, nearly continuous except for small semi-circular gap 2 (wide) × 1 mm (deep) where traversed by funicle; abscission layer between septa with well-developed spongy layer except absent at beak, vascular strand single or bifurcate; columella (carpophore) 18–20 mm long, proximally (where traversing pericarp at fruit base) rounded, distally (where confluent with septa) trigonous, distal part alate with jagged wing extending to 2 mm from central axis; funicle erect, slender, 2 × 0.3 mm. *Seeds* ellipsoid, apically (hilar end) rounded, basally flattened to slightly depressed, 8.8–9.7 (long) × 6.6–7.5 (wide; lateral-lateral) × 6.6–7 mm (deep; raphe-antiraphe), ventral face longitudinally traversed by narrow raised raphe 0.2 mm wide; seed coat ca 0.1 mm thick, testa thin, dry, uniformly dark brown; exotegmen mechanical, uniseriate palisade layer of elongate thick-walled cells, cells 3–4× shorter at apex versus bottom and sides, cell orientation vertical, inclined, or curved depending on location; caruncle absent; seed contents separated from mechanical coat by thin spongy layer except at base where solidly attached; endosperm yellowish, fleshy, slightly oily, forming layer up to 1 mm thick around central flattened ellipsoidal pocket containing embryo with cotyledons adhering to side of pocket; embryo type spathulate fully developed, embryo straight, extending most of seed length; cotyledons flat, elliptic, 4.5 × 3 mm, thin (ca 0.1 mm), apex broadly rounded, base subcordate, prominulous central vein that is distally branched; hypocotyl-radicle (stalk sensu Martin 1946) 2 × 1 mm, laterally slightly flattened.

#### Etymology.

The genus is combined from “*Inca*” (as *Inka*, Quechua for “ruler” or “lord”) referring to the indigenous Inca people and pre-Columbian empire that was centered in Cusco and encompassed much of the range of this taxon, and “*dendron*” (Greek) referring to tree, which is the habit of the plant. Some localities occur near the Trocha Unión, an ancient Inca path. The specific epithet is from “*Esser*”, the surname of Hans-Joachim Esser (Botanische Staatssammlung München, Germany) and honors this expert on Hippomaneae who has contributed much to our understanding of the tribe and Euphorbiaceae in general.

#### Distribution, life history, and ecology.

*Incadendron* is known from three well-separated clusters of localities (hereafter referred to as Cóndor, Manu, and Oxapampa populations) on the eastern slopes of the main Andean mountain range in Peru and Ecuador, where it occurs in wet montane forests at 1800–2400 m elevation (Fig. 2). The extent of discontinuity in its range is presently unclear due to the floristically poorly known nature of the intervening areas, and it should be looked for in similar habitats between the three populations. There are minor vegetative differences including leaf apex variation with most tips distinctly acute versus more rarely rounded (i.e., *Neill & Kajekai 16620*, *Monteagudo et al. 16929*), and a larger-leafed collection (i.e., *Monteagudo et al. 4458*). The differences exist within the populations and presently do not suggest differentiation worthy of taxonomic recognition.

Detailed field observations were made in the tropical montane cloud forests of the Kosñipata Valley in Manu National Park (Parque Nacional del Manu), which contains the southernmost part of the known range of *Incadendron*. The general site characteristics are well documented (see Girardin et al. 2010, Farfan Rios 2011, Feeley et al. 2011, Rapp et al. 2012) as part of intensive forest monitoring using permanent forests plots established by the Andes Biodiversity and Ecosystem Research Group (ABERG, http://www.andesconservation.org/) along an elevational gradient (i.e., Kosñipata transect) from the Andes to the Amazon. *Incadendron* has been found (i.e., *Farfan et al. 1049*, *1090*, *1131*) in the cloud immersion zone between 1800–2250 m at the study site. The substrate where the tree was collected is granite between 1800–2000 m, and shale at 2250 m. The soils below the thick organic surface layer are relatively poor in nutrients. At the elevations where found, *Incadendron* is among the taller components of the forest and its habit is a canopy tree with a spreading crown. The maximum height observed was 21.2 m, and maximum tree diameter at breast height (dbh) was 56.7 cm. When cut, the thin trunk bark has a cream-yellowish color with abundant white latex. Mean tree growth (diameter increment) at the study site was 4.02 mm yr^-1^ ± 0.90 (95% CI). Mean wood density is 0.55 g/cm^3^ ± 1.18 (95% CI), based in field sampling. The highest population density in the network of one hectare plots was found at 2000 m of elevation, with 30 adult individuals/ha (≥10 cm dbh), making it the ninth most abundant tree in that plot (Parcela VII). The main associated species include *Alzatea verticillata* Ruiz & Pav. (Alzateaceae), *Cyathea lechleri* Mett. (Cyatheaceae), and *Ilex villosula* Loes. (Aquifoliaceae). The Euphorbiaceae diversity for the tropical montane forests of this region includes nine genera, of which there is notable species-richness in *Alchornea* Sw. (Farfan-Rios et al. 2015). The closet relatives of *Incadendron* (i.e., other members of Euphorbioideae) among these nine genera include *Sapium* spp. and *Pseudosenefeldera inclinata* (Müll. Arg.) Esser, with the latter occurring at lower elevations. The basal marginal leaf glands of *Neill & Kajekai 16620* from the Cordillera del Cóndor are overgrown by unusual clusters of tiny, 0.1 mm diameter black globules that are fruiting bodies of a likely ascomycete fungus. While epiphyllous fungal growth such as mold growing on glandular secretions is to be expected, these unusual fruiting structures are very different and deserve further study.

#### Phenology.

The trees are evergreen, with an observed flowering season during July–September and fruiting during August–November. Herbarium collections also indicate a spring reproductive period of January–April for the Manu and Oxapampa populations. Fruits can be abundant, they turn brown when mature (Fig. 3G–H), and due to their large, heavy nature become pendulous on the relatively long infructescence axes. They are subject to predispersal seed predation by Lepidoptera, based on caterpillar remains recovered from inside fragmented fruits of *Monteagudo & Ortiz 4605*. In developing fruits these moths (likely members of Phycitinae, Pyralidae: Pyraloidea; A. Solis, personal communication) hollow out the seeds, which have well-developed endosperm, and leave holes (1.75 mm dia.) in the mericarp septa and seed coats (Fig. 3I–J). Plant defenses to deter herbivory would appear to be high in *Incadendron* due to latex, and the thick, lignified pericarp. Seed predation by specialist moths is well known for other Hippomaneae including in *Mabea* Aubl., where oviposition occurs early in fruit development when the pericarp is thin and soft (De Steven 1981). One young *Incadendron* fruit (4 mm dia.) on *Monteagudo & Ortiz 4605* (US) has what appears to be an oviposition hole at the top of the ovary that is likely a weak spot into the interior.

#### Conservation status.

Following the criteria and categories of IUCN (2012), *Incadendron esseri* is given a preliminary status of Vulnerable (VU) under geographic range criteria B2 area of occupancy <2000 km^2^ (B2a, known to exist at no more than 10 locations; B2b, continuing decline projected). Threats to this taxon in the Cordillera del Cóndor include mining for the underlying silica sand. Parts of its Peruvian range are protected within the Parque Nacional Yanachaga Chemillén and Parque Nacional del Manu.

#### Additional collections.

**ECUADOR. Zamora-Chinchipe**: Yantzaza Cantón. Cordillera del Cóndor region. Río Machinaza watershed, east of Los Encuentros, in and near a 0.25-ha forest inventory plot, tree #1477 in Aurelian Plot #6, La Zarza mining concession of Kinross Aurelian Corp., about 1.7 km southeast of and 500 m above Las Peñas camp, 03°47'50"S 78°29'05"W, 1840 m, 30 Jun 2009 (fl), *D. Neill & C. Kajekai 16620* (MO, US); [same locality], tree #1362 in Aurelian Plot #6, 1840 m, 30 Jun 2009 (fl), *D. Neill & C. Kajekai 16622* (MO, US); [same locality], tree #1477 in Aurelian Plot #6, 1840 m, 30 Jun 2009 (fl), *D. Neill & C. Kajekai 16646* (MO, US). **PERU. Cusco**: Prov. Paucartambo, Kosñipata, Trocha Unión, km 13, Parcela VIII, subparcela 16, árbol 706, 19L 0222887, UTM 8553630, 1835 m, 19 Aug 2003 (fl, fr), *W. Farfan et al. 1049* (CUZ, F, HUT, MO, USM, DAV; cited as *Garcia et al. 1049* in Farfan-Rios et al. 2015); Trocha Unión, km 10, parcela VI, subparcela 2, árbol 74, 19L 0221737, UTM 8552556, 2295 m, 4 Sep 2003 (sterile), *W. Farfan et al. 1090* (CUZ, MO, USM, WFU); Trocha Unión, km 11, parcela VII, subparcela 3, árbol 105, 19L 0222622, UTM 8553538, 2000 m, 9 Sep 2003 (fl, fr), *W. Farfan et al. 1131* (CUZ, MO, USM, WFU); Prov. Paucartambo, Callanga, 19L 196221, UTM 8578219, 2245 m, 13 Sep 2007 (sterile), *W. Farfan et al. 3635* (USM, WFU); Callanga, 19L 196364, UTM 8579065, 2110 m, 14 Sep 2007 (sterile), *W. Farfan et al. 3696* (USM, WFU). **Pasco**: Oxapampa, Distrito Oxapampa, Parque Nacional Yanachaga Chemillén, cercanías del Refugio el Cedro, 10°32'S, 75°21'W, 2240 m, 27 Nov 2002 (fl), *A. Monteagudo et al. 4458* (MO, US); Parque Nacional Yanachaga Chemillén, cercanías del Refugio el Cedro, 10°32'S, 75°22'W, 2200–2400 m, 6 Feb 2003 (fl, fr), *A. Monteagudo et al. 4484* (MO, US); Parque Nacional Yanachaga Chemillén, camino del Refugio al Abra La Esperanza, 10°31'S, 75°20'W, 2400 m, 8 Mar 2003 (fr), *A. Monteagudo & G. Ortiz 4605* (MO, US); Parque Nacional Yanachaga Chemillén, Refugio Abra Esperanza y sus alrededores, 10°31'55"S 75°20'59"W, 2786 m, 23 Apr 2009 (fr), *M. Cueva 592* (MO, US); Parque Nacional Yanachaga Chemillén, Estación Biológica San Alberto, Refugio El Cedro, 10°32'20"S 75°20'14"W, 2731 m, 11 Feb 2012 (fr), *R. Vásquez & L. Valenzuela 37638* (MO, US); Localidad Grapanazú, 10°29'34"S 75°23'28"W, 2288 m, 22 Nov 2012 (young fr), *R. Vásquez 38201* (MO); Distrito Huancabamba, zona de amortiguamiento del Parque Nacional Yanachaga Chemillén, al borde de las chacras y pastizales cercanías de la casa del Señor Orlando Quispe, 10°16'38"S 75°31'06"W, 1894 m, 24 Jul 2008 (young infl), *A. Monteagudo et al. 16929* (MO, US).

#### Discussion.

Specimens of *Incadendron* mostly were tentatively identified by collectors either as *Sapium* Jacq., due to similarities in coriaceous leaves and glandular, spicate inflorescences (Fig. 3A, D), or as *Micrandra* Benth. (e.g., Farfan-Rios et al. 2015), due to their shared unusually large fruits and white latex. *Sapium* notably differs in its bistaminate flowers and red-arillate seeds, and *Micrandra* is florally very different as a member of subfamily Crotonoideae. Within the context of the generic key to South American Hippomaneae in Athiê-Souza et al. (2015), *Incadendron* would group with *Sebastiania* Spreng. A comparison of select genera and distinguishing characters is given in Table 1. These genera are the most morphologically and geographically similar to *Incadendron* but are not necessarily its closest relatives, which are presently unclear. *Senefelderopsis* Steyerm., however, may have a closer relationship as suggested by similar fruit structure (see below), biogeographic ties between the Andean cordilleras and Guiana Shield (Berry and Riina 2005), and isolated phylogenetic placement in the same diverse subclade H1 of Hippomaneae (Wurdack et al. 2005).

*Incadendron* presents a unique combination of rare characters (discussed below) within Hippomaneae including sheathing stipules, conduplicate ptyxis, leaf margins entire and with unusual glands, and large, woody fruits. None of these characters appears phylogenetically very informative because they are autapomorphic or clearly homoplasious when considered in the context of molecular phylogenies (i.e., Wurdack et al. 2005, Wurdack, unpublished). Thus, while the rare character combination serves well to distinguish *Incadendron* and indicates a degree of morphological disparity deserving of generic recognition, it does not inform on relationships nor provide much insight into how such characters evolved. Major floral features that are variable in Hippomaneae have broadly distributed, likely plesiomorphic, states in *Incadendron*, including terminal and unbranched inflorescences, single pistillate flower per bisexual inflorescence, staminate cymules that are multiflowered and glandular, and 3-merous pedicellate staminate flowers with free stamens.

Pollen morphology and ultrastructure are remarkably diverse across Euphorbiaceae, but are less variable in Euphorbioideae, and Hippomaneae in particular (Punt 1962, Park and Lee 2013). The pollen of *Incadendron* resembles that of other hippomanoids in being tricolporate with a perforate exine (Fig. 4C–D). The seed structure of *Incadendron* is also similar to that of other Hippomaneae (Martin 1946, Tokuoka and Tobe 2002). Its seed coat (Fig. 3L) with a thin testa of collapsed cells and palisade-like mechanical exotegmen of elongate, pitted, thick-walled cells resembles that of exotegmic genera across the entire family. Depending on exact location, the pallisadal cells vary in length (3–4× shorter at apex versus bottom and sides) and orientation (vertical, inclined, or curved). Embryos generally deserve more careful description and study given their diversity in Euphorbiaceae, although most are variants of the spathulate fully developed type (Martin 1946). The embryo of *Incadendron* agrees with this generalization (see Description for details, based on one intact seed) and in particular resembles that of other Hippomaneae including *Senefelderopsis croizatii* Steyerm. (examined here, *Maas et al. 5828*, US), which has a slightly longer hypocotyl-radicle axis at 3 × 1 mm (versus 2 × 1 mm) and thicker cotyledons at 0.5 mm (versus 0.1 mm).

#### Unusual morphological features of *Incadendron*.

**Figure 1.**
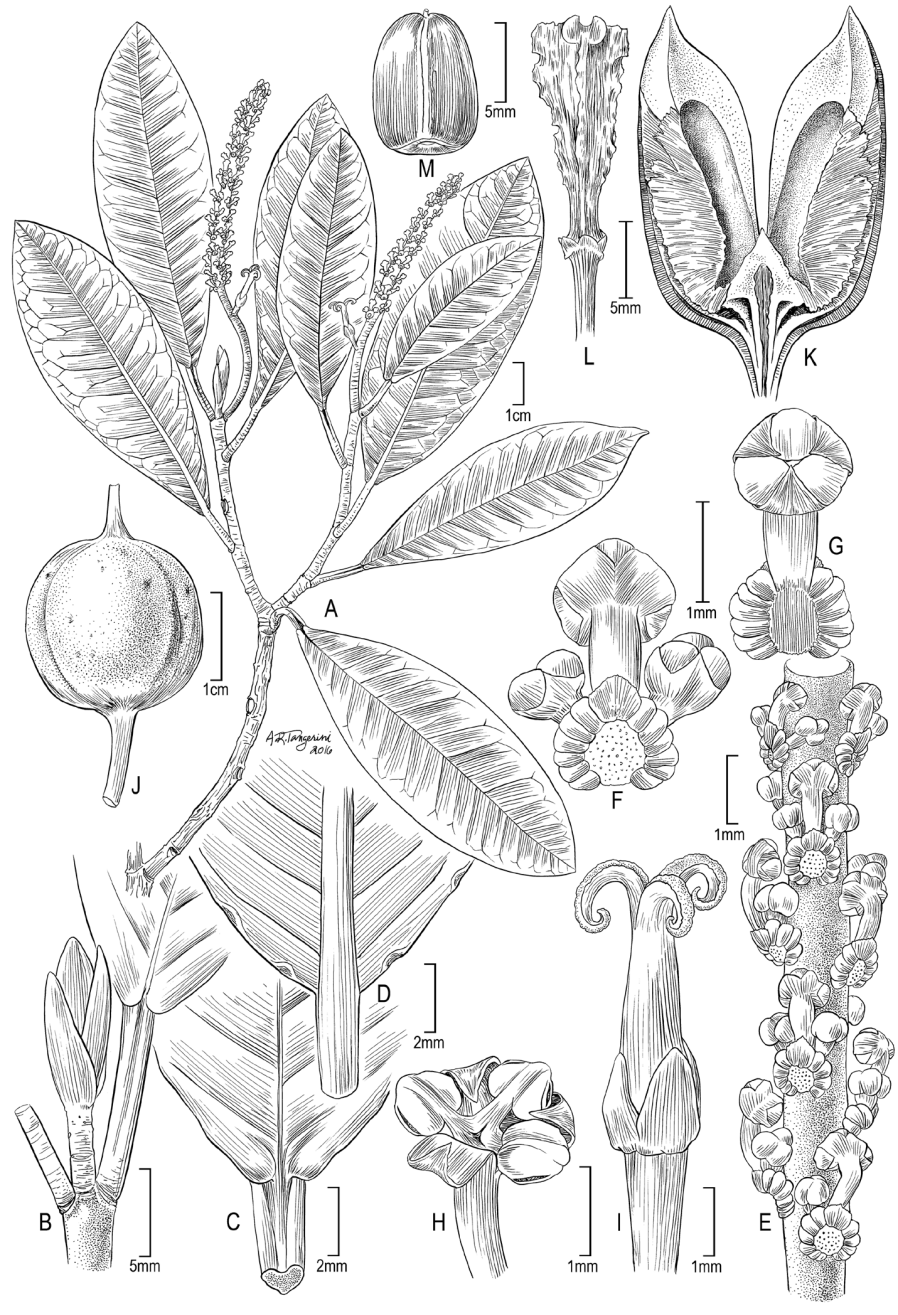
Illustration of *Incadendron esseri*. **A** Habit **B** Shoot tip **C** Leaf base (adaxial) **D** Leaf base and marginal glands (abaxial). **E** Staminate subinflorescence **F** Staminate cymule (distal view) **G** Staminate cymule (proximal view, without lateral buds) **H** Staminate flower **I** Pistillate flower **J** Fruit **K** Mericarp valve **L** Columella **M** Seed (ventral face). (Source: **A–G** *Calatayud et al. 4711*, MO; **H–I** *Monteagudo et al. 4458*, US; **J** *Vásquez & Valenzuela 37638*, MO; **K–M** *Monteagudo & Ortiz 4605*, US).

The entire hippomanoid clade (sensu Wurdack et al. 2005) is considered here for the purposes of broad discussion rather than restricted to paraphyletic Hippomaneae. This broad grouping is reflected in the nomenclaturally problematic (see Esser 2012) Hippomaneae s.l. of Webster (2014). Stipules in the hippomanoids are typically small, scale-like (Fig. 5D) or absent. Conspicuous, sheathing stipules are relatively rare and in addition to *Incadendron* (Fig. 1B) also characterize *Conosapium* Müll. Arg., *Homalanthus* A. Juss. (Fig. 5A), *Hura* L., *Neoshirakia* Esser, and *Pachystroma* Müll. Arg. While functionally these large stipules are similar in conferring additional protection to the shoot apex, they differ in morphological details and likely are convergent size increases. The stipules in *Incadendron* are large, sheath the terminal bud, unusually stout in keeping with the coriaceous leaves, deciduous, and centrally attached such that an elliptic scar remains after abscission (Fig. 3E). The buds are internally mucilaginous. Serial sections of a single bud show a distinct palisade-like cell layer on the inner surfaces of the stipules that is the likely source of this secretion (Fig. 4H–I). This layer appears to differentiate late in development as it is present in the outer stipules but not on the enclosed next younger pair of the bud (Fig. 4H). The inflorescence bracts resemble a smaller version of the stipules (rarely with a few subtending glands of the type found with the staminate cymules) and are also deciduous, leaving large scars (Figs 3F, 4B). Conspicuous sheathing stipules in other taxa are mostly thinner except in *Pachystroma*, and may be centrally attached, leaving elliptic scars (*Homalanthus*, *Hura*, *Neoshirakia*) or broadly attached along their entire base, leaving semi-circumferential scars (*Conosapium*, *Pachystroma*).

Leaf folding (ptyxis) is variable but poorly studied for Euphorbiaceae in general (Cullen 1978). Based on recent surveys (K. Wurdack, personal observations) *Incadendron* appears unique in the hippomanoids in having conduplicate ptyxis (Fig. 4G). In bud and early expansion after stipule abscission, the halves of the blade are folded tightly together along their adaxial surface and the leaf in transverse section shows no curling at the edges. After the blade halves spread open, the margins recurve before hardening and finally at maturity the halves assume a flattened aspect with a slightly revolute margin (Fig. 3A, C). The terminal buds are flattened (Fig. 3E, 4G), which likely reflects the conduplicate nature of the enclosed developing leaf blades, although this point needs further study with fresh material. Other hippomanoids have varying degrees of developing leaf blade curvature including tightly rolled scrolls, rolled edges, or gently curved loops that span involute (e.g., *Homalanthus*, Fig. 5B) to supervolute-curved ptyxis (e.g., *Senefelderopsis*). Cullen (1978) indicated the hippomanoid *Excoecaria cochinchinensis* Lour. (as *E. bicolor* [Hassk.] Zoll. ex Hassk.) was conduplicate. Our observations on living (Fig. 5C) and herbarium material indicate its glandular-toothed margin is inwardly curled and the ptyxis is more accurately described as a conduplicate-involute intermediate. The hippomanoids contain a wide diversity of petiolar and leaf gland form and position. *Incadendron* has no acropetiolar or embedded laminar glands but it has glands along the leaf margin, which are best developed (i.e., consistent in presence and largest in size) near the leaf base. Curling of the leaf edge with age shields these basal glands (Fig. 3C), although the scattered more distal glands remain exposed. The marginal glands (Fig. 4E) are not associated with teeth or setae and are similar in morphology to those found on *Gymnanthes schottiana* Müll. Arg. Hippomanoid leaf margins typically have regular teeth or marginal setae, and are more rarely similar to *Incadendron* or entire without any associated glands. The abaxial leaf surface of *Incadendron* is finely striate, but the stomata are not shielded or sunken (Fig. 4F). Some high elevation hippomanoids have micro-papillose surfaces and concealed stomata (e.g., *Dendrothrix* Esser, *Senefelderopsis*).

Inflorescences in the hippomanoids are axillary and/or terminal. In *Incadendron* the inflorescence is terminal but appears distinctly leaf-opposed due to near simultaneous development of both the inflorescence and adjacent leaf, coupled with the start of renewal shoot growth from the axillary bud before flowering is finished (Fig. 3E). Axial growth and orientation in *Incadendron* branches is not deflected by inflorescence development and gives little hint of being sympodial by substitution. Specimens of *Incadendron* show twinned branching (Fig. 1A, 3A), due to the occasional growth of additional leaf-axil accessory buds close to the growing branch tips. Most accessory buds, even in older parts of the shoot system, remain as barely visible meristems.

Large fruits, ≥2 cm in diameter are relatively rare among the hippomanoids (i.e., *Incadendron esseri*, *Duvigneaudia inopinata* [Prain] J. Léonard, *Hippomane mancinella* L., *Pachystroma longifolium* [Nees] I.M. Johnst., *Sebastiania obtusifolia* Pax & K. Hoffm., and a few species each of *Algernonia* Baill., *Excoecaria* L., *Hura* L., *Mabea* Aubl., *Ophthalmoblapton* Allemão, *Sapium*, *Senefeldera* Mart., *Senefelderopsis*, and *Shirakiopsis* Esser), which otherwise typically have fruits less than half that size. Among the likely correlates of increased fruit size are larger seeds (see size variation in Tokuoka and Tobe 2002), thickened pericarps, indehiscence, and being borne singly (rarely two). Especially noteworthy is the combination of a dehiscent fruit with a thick pericarp (≥3 mm), which is rare among the big-fruited species. These generalizations do have exceptions such as thin walls in inflated fruits (i.e., *Excoecaria bussei* [Pax] Pax), or multiple fruits in large-fruited taxa with robust or compound inflorescences (e.g., *MabeaSenefeldera*, *Senefelderopsis*). *Incadendron* exhibits some of these large-fruit trends including seed size, pericarp thickness, and being borne singly.

The fruits of *Incadendron* most closely resemble those of *Senefelderopsis*. In addition to large size, they share many structural features including a thick pericarp with an exceptionally thick woody mesocarp (≥3 mm), sharp woody apex, well-developed septa, mericarp valves connected with a basal triangle, and a thin funicle. These features individually (or in various combinations) occur in other genera, but the thick mesocarp is exceptional among the large-fruited, dehiscent species. The mesocarp of *Incadendron* has a prominent dorsal-suture lip, has non-vascularized raised ridges that follow underneath major exocarp venation, and is stratified with a darker layer of different structure lining the locule (Fig. 3M); these features are to varying degrees shared with *Senefelderopsis* (Fig. 3N). In both *Incadendron* and *Senefelderopsis* the robust septa dividing the locules are lignified and nearly complete except for a small apical gap (Fig. 3J–K, M–N) to accommodate the funicle and its attachment to the placenta. Many other hippomanoids differ in having thin, poorly lignified septa that become destroyed during dehiscence, and/or septa that are incomplete leaving a large gap descriptively called a “C-shaped cut” (Athiê-Souza et al. 2015). Fruit vascular variation, especially septal strand number, has been considered potentially informative at the generic level in Hippomaneae (Esser et al. 1997, Esser 2001), but can be difficult to observe and its functional significance is not well understood. The septal vascular strand numbers are very similar between *Incadendron* (single or bifurcating) and *Senefelderopsis* (single or rarely bifurcating). The seemingly delicate, suspended nature of the relatively large seeds in the locules of both genera is unusual. Their thin, nearly erect funicles differ in length, which allow the seeds of *Incadendron* to be further displaced downward in the locule from the point of attachment to the columella as compared with *Senefelderopsis* (Fig. 3I–K). Many other hippomanoid taxa have better support for their seeds either through greater filling of the locule cavity by the seed that leaves little free space around it, and/or through more robust attachment to the columella via a short and thickened funicle or fusion along the raphe. The fruit and locule shape differ slightly between the genera in being subglobose in *Incadendron* versus elongate and more trigonous in *Senefelderopsis* (Fig. 3J–K, M–N). In addition, the exocarps (not examined anatomically and topographically defined here as the more or less removable outer layer) differ in gross structure with prominent vasculature at the interface between the loosely attached exocarp and woody mesocarp in *Incadendron*, likely supporting greater fleshiness when fresh. In *Senefelderopsis* there is little exocarp vascularization and tight adherence via a stiff, porous layer of apparently sclerified cells that is best developed in *S. chiribiquetensis* (R.E. Schult. & Croizat) Steyerm. Although there are considerable morphological differences between *Incadendron* and *Senefelderopsis* (see Table 1), the striking fruit similarities may have special significance for indicating a closer relationship between the genera.

**Figure 2.**
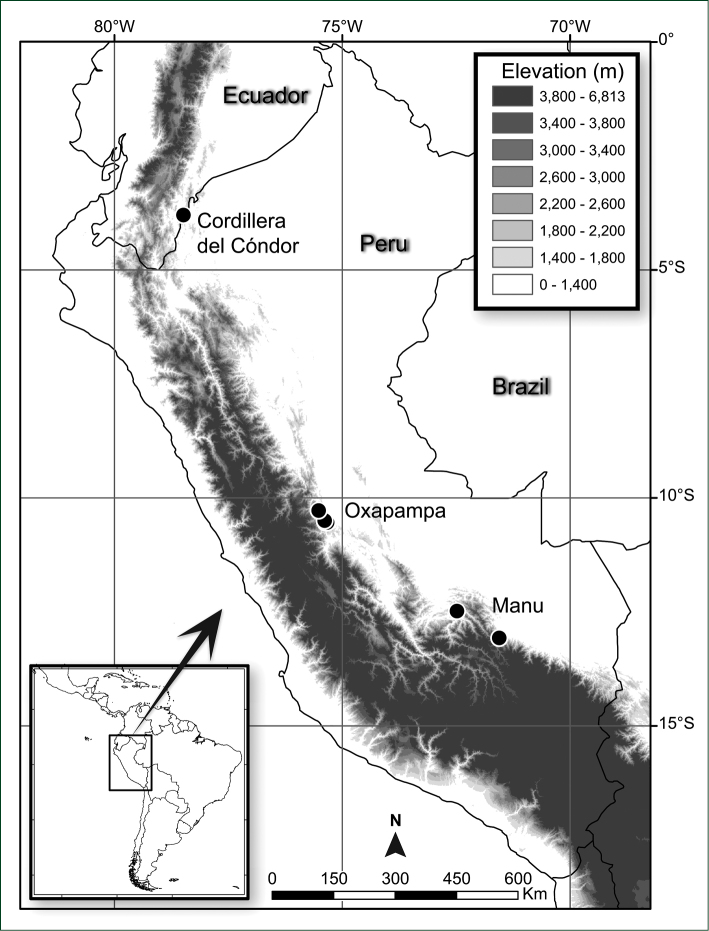
Distribution map of *Incadendron esseri*.

**Figure 3.**
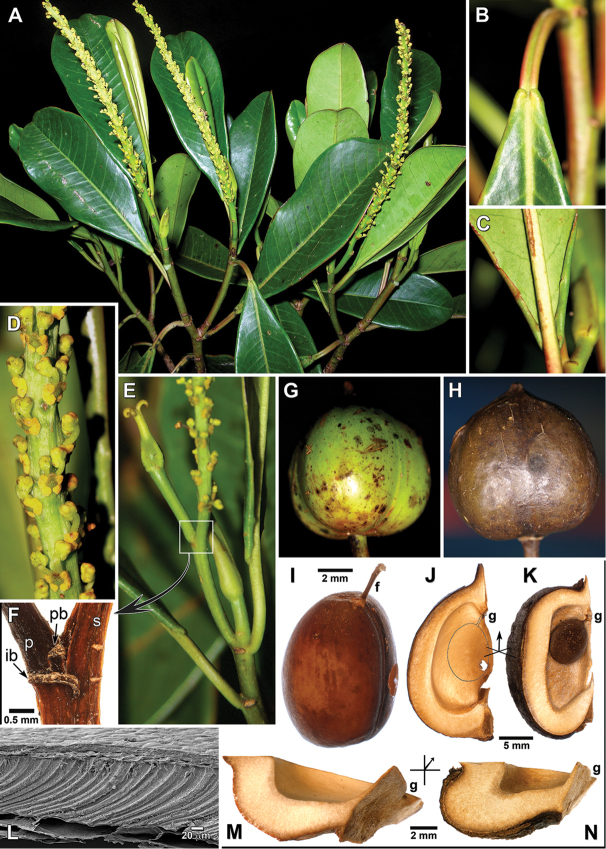
Morphology of *Incadendron* (**A–J, L–M**) and *Senefelderopsis* (**K, N**). **A** Habit, with paired branching and staminate inflorescences; note latex at damaged nodes **B** Leaf base (adaxial) with basal lobes **C** Leaf base (abaxial) with curled glandular margin **D** Staminate inflorescence with cymules subtended by glands; central flowers abscised leaving two lateral buds per cymule **E** Branch tip showing leaf-opposed inflorescence and stipule-enclosed renewal shoot **F** Summit of peduncle showing bract scars **G** Nearly mature green fruit **H** Mature brown fruit **I** Seed with funicle; holes in I & J come from insect predation **J** Mericarp valve with outline of seed position **K** Mericarp valve with a seed; funicle obscures gap **L** Seed coat, transverse view (SEM) **M** Pericarp profile and top half of valve (exocarp removed) **N** Pericarp profile and top half of valve. (Abbreviations: f = funicle, g = gap, ib = inflorescence bract scar, p = pistillate, pb = pistillate bract scar, s = staminate. Orientation of **M–N** relative to **J–K** shown by diagrams where x-y = plane of cross section, z = apically pointing arrow. (Source: Incadendron, **A–E, G–H** *Farfan et al. 1049*, *1131*; **F** *Farfan et al. 706*, MO; **I–J, M** *Monteagudo & Ortiz 4605*, US; **L** *Monteagudo et al. 4484*, US. *Senefelderopsis croizatii*, **K, N** *Radosavljevic 296*, US).

**Figure 4.**
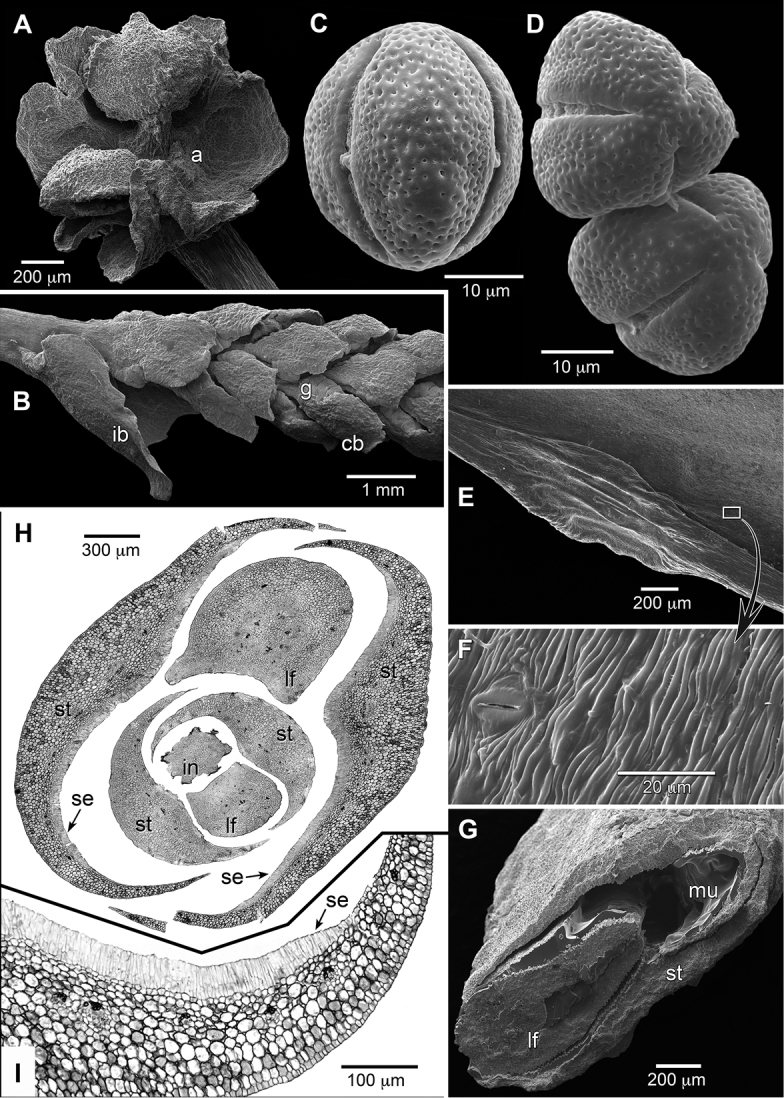
Micromorphological and anatomical features of *Incadendron*. **A** Staminate flower with one anther removed, showing short filaments and basally connate sepals **B** Young inflorescence, showing inflorescence bract and staminate cymules subtended by glands and bracts **C** Pollen; mesocolpium-centered equatorial view of whole grain **D** Pollen; slightly oblique polar view of whole grains **E** Abaxial laminar surface with marginal gland **F** Abaxial laminar surface closeup, showing striate micro-sculpturing and stomata **G** Fractured shoot tip, showing stipule surrounding young leaf with conduplicate ptyxis **H** Anatomical cross section of shoot apex, showing central terminal inflorescence surrounded by nested series of two developing leaves with subtending sheathing stipules (composite tiled image) **I** Closer view of anatomical cross section of stipule, showing mucilage-secreting cell layer. (Abbreviations: a = site of attachment of missing anther, cb = cymule bract, g = glands, ib = inflorescence bract, in = inflorescence, lf = leaf, mu = dried mucilage, se = secreting cells, st = stipule. **A–G** imaged with a Zeiss EVO MA15 SEM at 10–12 kV after sputter coating with 25 nm of Au/Pd; SEM samples untreated and directly mounted from dried herbarium specimens; pollen from dehiscing anthers. **H–I** from paraffin-embedded, rehydrated herbarium specimens; 7 μm sections stained with iron-mordanted safranin O and celestine blue B; imaged with a Zeiss Universal. Source: **A, C–D** *Monteagudo et al. 4458*, US; **B** *Neill & Kajekai 16622*, US; **E–I** *Neill & Kajekai 16646*, US).

**Table 1.** Comparison of *Incadendron* with the morphologically most-similar neotropical genera. Based on primary observations with supplements from Kruijt (1996) and Esser (1995, 2001). The circumscription of *Sebastiania* is controversial leading to some uncertainty in the breadth of character states.

Character	*Incadendron*	*Senefelderopsis*	*Sapium*	*Sebastiania*
Stipules	Lanceolate, sheathing, 10–13 × 3–4 mm, deciduous leaving elliptic scar	Lanceolate, 2.5–5 × 0.5–0.8 mm, deciduous leaving trigonous scar	Triangular, usually <2 × 2 mm but up to 8 mm long, persistent (rarely tardily deciduous)	Lanceolate, small, persistent
Indument	Absent	Present; multicellular, uniseriate	Absent	Usually absent; multicellular, uniseriate in some potential segregates (e.g., *Sebastiania vestita* Müll. Arg.)
Leaf features	Coriaceous; glands along margin; margin entire; ptyxis conduplicate	Coriaceous; large glands at base and rimmed laminar glands; margin entire; ptyxis supervolute-curved	Usually coriaceous; petiolar glands 2 (0 or 4), laminar glands absent; margin entire, glandular or toothed; ptyxis involute	Membranous to subcoriaceous; glands absent or rimmed glands near base; margins usually minutely toothed; ptyxis involute
Inflorescence structure	Terminal, simple thyrse; 0–1 pistillate proximal, staminate distal in numerous 3-flowered cymules; cymule bract glands 4–5 per side, discoid; bracteoles absent	Terminal, compound thyrse; 0–2 pistillate proximal per branch, staminate distal in numerous 5–10 flowered cymules; cymule bract glands 1 per side, fleshy and elongate; bracteoles present	Terminal or axillary, simple thyrse; 0–15 pistillate proximal, staminate distal in numerous 2–16 flowered cymules; cymule bract glands 1 per side, elliptic, flattened; bracteoles present	Terminal or axillary, simple thyrse; 0–5 pistillate proximal, staminate distal in numerous 1–3(7)-flowered cymules; cymule bract with 1 discoid gland to large segmented glandular mass per side; bracteoles usually absent
Staminate flowers	Stamens 3; sepals 3, connate at base	Stamens 3–5; sepals 3, connate at base	Stamens 2; sepals 2, connate at base to 2/3 of length	Stamens 3; sepals 3, connate at base
Fruits	Large; pericarp thick, woody	Large; pericarp thick, woody	Small to large; pericarp woody to leathery, usually thin	Small to large; pericarp thin

**Figure 5.**
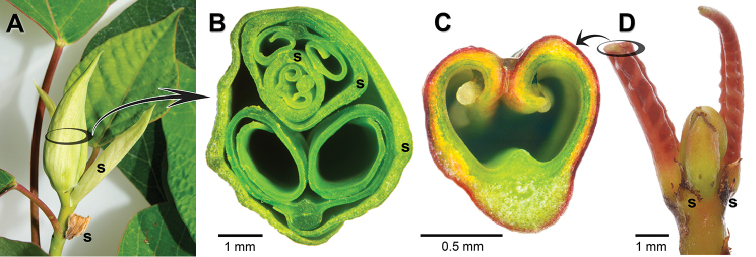
Hippomaneae shoot tips showing stipule and ptyxis variation. **A** *Homalanthus nutans* (G. Forst.) Guill. with large, deciduous, sheathing stipules protecting shoot tip **B** Transverse view of *H. nutans* shoot tip, showing nested series of three developing leaves with scroll-like lamina (ptyxis involute), each surrounded by a pair of sheathing stipules **C** Transverse view of *Excoecaria cochinchinensis* Lour. young leaf showing slightly inrolled lamina (ptyxis conduplicate-involute) **D** *E. cochinchinensis* shoot tip with small persistent stipules. (Abbreviation: s = stipule. Source: Freehand sections of fresh tissues grown in Department of Botany greenhouses).

## Supplementary Material

XML Treatment for Incadendron

XML Treatment for Incadendron esseri
